# Crustaceans from antipatharians on banks of the northwestern Gulf of Mexico

**DOI:** 10.3897/zookeys.457.6280

**Published:** 2014-11-25

**Authors:** Mary K. Wicksten, Marissa F. Nuttall, Emma L. Hickerson

**Affiliations:** 1 Biology, Texas A&M University, College Station Texas U.S.A. 77843-3258; 2 Flower Gardens Banks National Marine Sanctuary, 4700 Avenue U, Galveston, Texas 77551 U.S.A.

**Keywords:** Anomura, Caridea, Cirripedia, Antipatharia, Gulf of Mexico

## Abstract

The stalked barnacle *Oxynaspis gracilis*, the chirostylid squat lobster *Uroptychus* sp., and the caridean shrimps Periclimenes cf. antipathophilus and *Pseudopontonides principis* have been collected at 68–124 m by a remotely operated vehicle (ROV) on banks in the northern Gulf of Mexico. These species inhabited six species of antipatharian hosts. *Pseudopontonides principis*, *Oxynaspis gracilis*, and *Uroptychus* sp. were not confined to a single host species. Except for *Oxynaspis gracilis*, collected by ROV in 2004–2005, these species have not been reported previously in the northwestern Gulf of Mexico.

## Introduction

**Figure 1.**
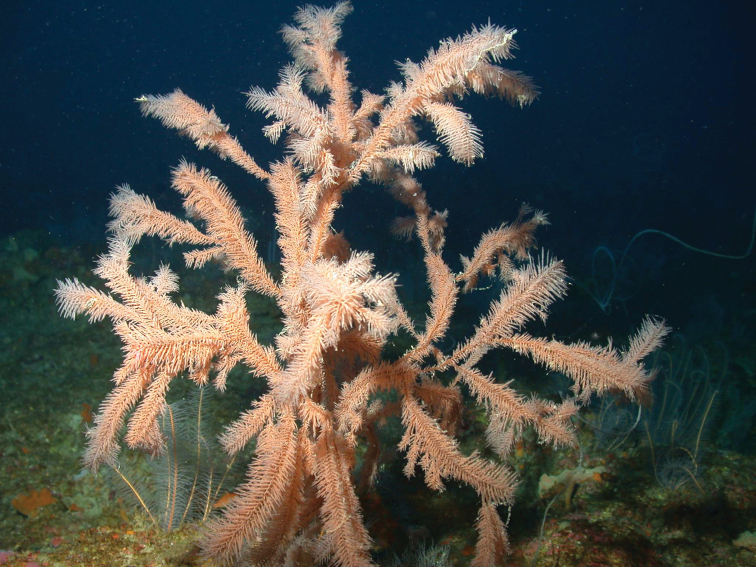
Deep reef habitat showing antipatharians *Tanacetipathes* sp. (foreground) and *Stichopathes* sp. (distant) among protruding arms of comatulid crinoids (West Flower Garden Bank, 80 m).

Antipatharians (Cnidaria: Anthozoa: Hexacorallia), commonly called black corals or wire corals, tend to inhabit deeper reef areas or cliffs in the greater Caribbean-west Atlantic region (Fig. 1). In the Gulf of Mexico, they rarely inhabit depths that can be reached by SCUBA divers. Boland (2005) reported *Plumapathes pennacea* (Pallas) from four oil platforms at 9.5-43.0 m at the following locations: 28° 00'N, 93°17'W; 27°8'N, 93°40'W; 27°46'N, 93°8'W; 27°46'N, 93°19'W; and at the East Flower Garden Bank buoy 6, 27°55'N, 93°36'W. One of us (MW) saw (but did not collect or photograph) a “wire coral” (*Stichopathes* sp.) at a depth of 33 m on West Flower Garden Bank (27°52.5'N, 93°49'W), 2 Sept. 2004.

Black corals have been documented to host many associated species, including crustaceans, polychaetes, mollusks, ophiuroids, and fishes (Totton 1923, Warner 1981, Humes 1990, Grange 1991, Pettibone 1991, Glasby 1994, Wirtz and d’Udekem-d’Acoz 2001, Castro et al. 2004, Molodtsova and Budaeva 2007, Tazioli et al. 2007). We have not found any records of antipatharian-associated fauna in the northwestern Gulf of Mexico. We here present records, depths and species associations of a cirripede and three decapod crustaceans that associate with black corals.

## Methods

MN collected specimens in the vicinity of the East and West Flower Garden Banks, Horseshoe Bank, 29-Fathom Bank, Rankin Bank, 28-Fathom Bank, Bright Bank, and Geyer Bank at depths ranging from 68.0 to 123.9 in 2011–2012 (Table 1). Figure 1 shows the natural habitat. Specimens were retrieved using a single function manipulator on a Phantom S2 remotely operated vehicle (ROV). When associated fauna were found, they were removed carefully from the black coral, photographed, and placed in separate containers with 95% ethanol. The photographs of the freshly collected shrimps are not sufficiently detailed for publication, but a photograph of *Uroptychus* sp., although the specimen was broken, is of sufficient detail to show the living color pattern.

**Table 1.** Material examined

Coordinates	Collection number	Depth, date	Number, host
*Oxynaspis gracilis*:			
East Flower Garden Bank:			
27°57.2'N, 93°36.0'W	DFH8-19A14, TCWC 2-8958	60 m, 3 Sept. 2004	2, on *Antipathes atlantica*/*gracilis*
27°57'N, 93°36'W	DFH11-3A, TCWC 2-9121	99 m, 13 Sept. 2005	3, on “*Antipathes* sp.”
West Flower Garden Bank:			
27°51.2'N, 93°49.2'W	DFH17-17C, TCWC 2-3663	82.4 m, 1 Aug. 2012	1, on *Phanopathes expansa*
27°53.9'N, 93°47.0'W	DFH17-25B, TCWC 2-3665	97 m, 1 Aug. 2012	1, on *Elatopathes abientina*
27°56.8'N, 93°37.4'W	DFH17-30C, TCWC 2-3666	87.8 m, 2 Aug. 2012	1, on *Antipathes atlantica*/*gracilis*
27°53.9'N, 93°27.0 W	PSBF2-18B, TCWC 2-3695	116.4 m, 22 Sept. 2012	2, on *Acanthopathes thyoides*
27°51.1'N, 93°26.3'W	PSBF2-19B, TCWC 2-3696	115.5 m, 22 Sept. 2012	2, on *Acanthopathes thyoides*
27°53.8'N, 93°19.6'W	PSBF3-2D, TCWC 2-3668	86.2 m, 26 Sept. 2012	1, on *Tanacetipathes barbadensis*
27°53.4'N, 93°15.6'W	PSBF3-4B, TCWC 2-3669	123.9 m, 26 Sept. 2012	1, on *Antipathes furcata*
27°52.2'N, 93°17.6'W	PSBF3-8B, not catalogued	89.1 m, 27 Sept. 2012	15, on *Antipathes atlantica*/*gracilis*
27°50.6'N, 93°3.6'W	PSBF3-12B, not catalogued	90.7 m, 28 Sept. 2012	2, on *Antipathes atlantica*/*gracilis*
27°50.3'N, 93°3.7'W	PSBF3-16B, TCWC 2-3698	86.2 m, 28 Sept. 2012	4, on *Antipathes atlantica*/*gracilis*
Horseshoe Bank:			
27°49.9'N, 93°3.37'W	PSBF3-17B, donated to California Academy of Sciences	87.8 m, 28 Sept. 2012	2, on *Antipathes atlantica*/*gracilis*
27°48.8'N, 93°41.5'W	PSBF1-3B, TCWC 2-2-3694	112.5 m, 24 Oct. 2011	4, on *Phanopathes expansa*
27°52.4'N, 93°42.3'W	PSBF3-10B, TCWC 2-3697	106.1 m, 25 Oct. 2011	2, on *Phanopathes expansa*
29-Fathom Bank:			
28°7.7'N, 93°28.9'W	PSBF1-13C, TCWC 2-3667	68 m, 26 Oct. 2011	1, on *Antipathes atlantica*/*gracilis*
*Uroptychus* sp.:			
West Flower Garden Bank:			
27°51.5'N, 93°49.7'W	DFH17-15B, TCWC 2-3636, one donated to U.S. National Museum	78.5 m, 1 Aug. 2012	2, on *Tanacetipathes thamnea*
27°51.3'N, 93°49.6'W	DFH17-16E, TCWC 2-3657	81 m, 1 Aug. 2012	1, on *Tanacetipathes tanacetum*
27°50.1'N, 93°51.3'W	DFH17-22B, TCWC 2-3658	120.3 m, 1 Aug. 2012	1, on *Acanthopathes thyoides*
Rankin Bank:			
27°55.2'N, 93°24.8'W	PSBF2-3C, sent to Kumamoto University	89.3 m, 11 Sept. 2012	2, on Tanacetipathes cf. paula
Periclimenes cf. antipathophilus:			
West Flower Garden Bank:			
27°50.9'N, 93°48.1'W	DFH17-20C, TCWC 2-3646	112.8 m., 1 Aug. 2012	1, on *Acanthopathes thyoides*
*Pseudopontonides principis*:			
West Flower Garden Bank:			
27°51.5'N, 93°49.7'W	DFH17-15C, TCWC 2-3648	78.5 m. 1 Aug. 2012	1, on *Tanacetipathes thamnea*
27°51.3'N, 93°49.6'W	DFH17-16B, TCWC 2-3649	81 m, 1 Aug. 2012	1, on *Tanacetipathes tanacetum*
27°51.3'N, 93°49.6'W 27°51.2'N, 93°49.2'W	DFH 17-16C, TCWC 2-3654 DFH 17-17B, TCWC 2-3650	81 m, 1 Aug. 2012 82.4 m, 1 Aug. 2012	1, on *Tanacetipathes tanacetum* 1, on *Phanopathes expansa*
East Flower Garden Bank:			
27°53.3'N, 93°36.8'W	DFH17-33D, TCWC 2-3651	90.9 m. 2 Aug. 2012	4, on *Tanacetipathes thamnea*
27° 53.3'N, 93°36.8'W	DFH17-33C, TCWC 2-3662	90.9 m, 2 Aug. 2012	1, on *Tanacetipathes thamnea*
Bright Bank:			
27°53.8'N, 93°19.6'W	PSBF3-2C, TCWC 2-3653	86.2 m, 26 Sept. 2012	1, on *Tanacetipathes tanacetum*
Rankin Bank:			
27°55.2'N, 93°24.8'W	PSBF2-3C, TCWC 2-3655	89.3 m, 19 Sept. 2012	1, on Tanacetipathes cf. paula

Antipatharian samples were identified using traditional morphological techniques (corallum branching mode, subpinnule branching patterns, spine morphology and size, and polyp size and distribution). Tissue was removed from a small section of each colony using a 50/50 sodium hypochlorite water solution and an ultrasonic cleaner, dried, and coated with gold-palladium using a sputter coater (70 mm target distance, 30 mA, 30 secs) to obtain scanning electron micrographs using a Hitachi TM3000 tabletop scanning electron microscope for analysis of skeletal morphology. MN identified specimens, with problematic species identifications confirmed by black coral taxonomist Dennis Opresko, Research Associate, United States National Museum of Natural History. Antipatharians recently have undergone taxonomic revision (Opresko 1972, 1974, 2001, 2004; Perez et al. 2005). We used currently accepted names when we were sure of the identification. Taxonomic confusion remains regarding the differences (if any) between *Antipathes atlantica* Gray and *Antipathes gracilis* Gray, hence in our records we record these species as *Antipathes atlantica*/*gracilis*. See Nuttall (2013) for further information on methods and the scope of the projects involved during collection.

Records of the material examined are given in Table 1. Host antipatharians species are not reported for many of the shrimps because they were loose from their hosts after collection. Because the two species are considered to be obligate associates of antipatharians, we are confident that they were living on antipatharians before collection. MW identified the carideans and most of the cirripedes. Stephen Gittings, United States National Oceanographic and Atmospheric Administration, identified the specimens of *Oxynaspis gracilis* Totten from DFH8-19A14.

Except as noted, the specimens were incorporated into the Biodiversity Research and Teaching Collection at Texas A&M University (formerly the Texas Cooperative Wildlife Collection, TCWC). A few specimens do not have catalog numbers because they are being used for further study. Two specimens of *Uroptychus* sp. were sent to Keiji Baba, Kumamoto University, Japan, for identification, and another specimen was donated to the collections of the U.S. National Museum of Natural History. Although originally thought to belong to *Uroptychus minutus* Benedict, 1902, these specimens now are considered to represent an undescribed species. Two specimens of *Oxynaspis gracilis* were donated to the California Academy of Sciences. The collections were part of the Deep Fish Habitat (DFH) and Potentially Sensitive Biological Features (PSBF) studies of the Flower Gardens Banks National Marine Sanctuary collected under permit number FGBNMS-2009-001.

## Results

**Figure 2.**
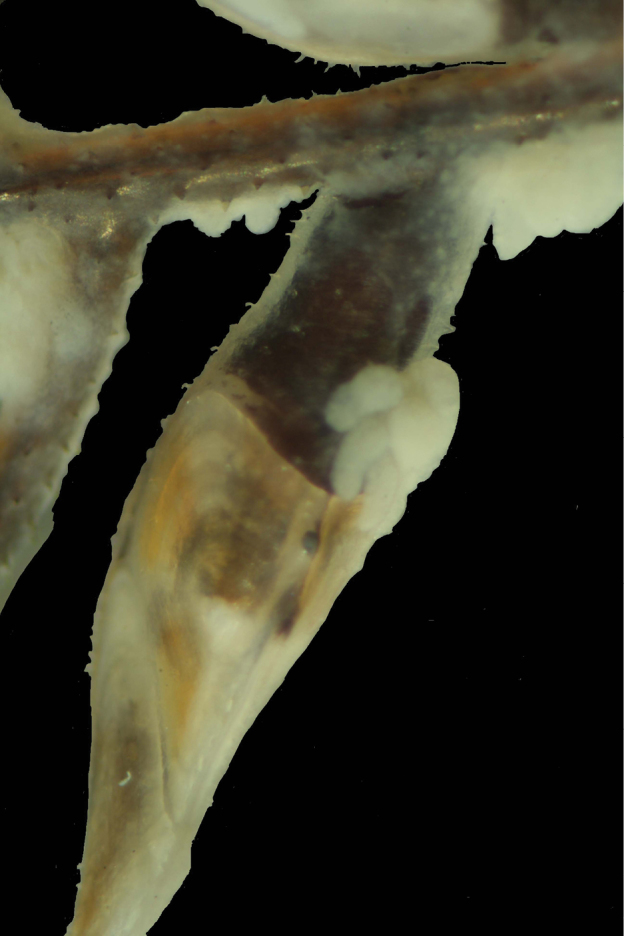
*Antipathes atlantica*/*gracilis* overgrowing *Oxynaspis gracilis*. (Horseshoe Bank, 112 m, sta. PSBF1-3B).

*Oxynaspis gracilis*, the black coral barnacle (Cirripedia, Lepadomorpha, Oxynaspididae) is a small (2–5 mm in total length), stalked species found attached to the axes of the corals. It was found on six species, including bottlebrush and fan-shaped species, at 68.0 to 123.9 m. The living tissue of the coral host may encrust the cirriped (Figure 2). This barnacle was collected previously at the East Flower Garden Bank (Table 1). Humann and DeLoach (2002) reported *Oxynaspis gracilis* as “occasional Caribbean” on *Plumapathes pennacea* (Pallas) (as *Antipathes pennacea*) and *Antipathes salix* Pourtalés. (P.A. McLaughlin, Western Washington University verified the identification of the cirripeds in their photograph). Gittings et al. (1986) did not report this species from the northern Gulf of Mexico in their guidebook, nor is it listed in the master crustacean species list for the United States and Canada (McLaughlin et al. 2005).

**Figure 3.**
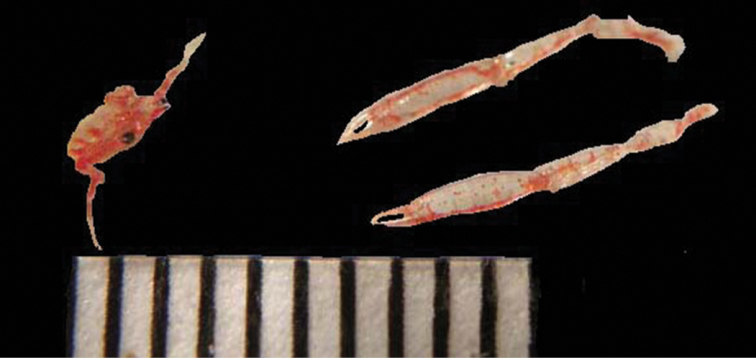
*Uroptychus* sp., freshly caught (West Flower Garden Bank, 79 m). Scale in mm.

*Uroptychus* sp. (Decapoda, Anomura, Chirostylidae) was found clinging to the main axes of *Tanacetipathes* spp. and the “sea fan” antipatharian *Acanthopathes thyoides*, (Pourtalés) at 78.5–120.3 m. Photographs of a freshly collected individual show that the cephalothorax was red, and the chelipeds translucent with a red lateral stripe on the propodus and carpus and red spots on the palm of the chela (Figure 3). An as yet unidentified specimen of *Uroptychus* sp. from southwestern Florida has a similar color pattern (D. Felder, pers. comm.)

Two shrimps (Decapoda, Caridea, Palaemonidae) were found on the antipatharians. *Pseudopontonides principis* (Criales), the wire coral shrimp, previously has been reported from the northeastern Gulf of Mexico and Curacao, Bonaire, and Puerto Rico in the Caribbean (Heard 1986, Humann and DeLoach 2002) on *Stichopathes leutkeni* (Brook) (as *Cirrhipathes leutkeni*), *Antipathes gracilis*, *Plumapathes pennacea*, and *Virgularia* sp. (Pennatulacea). In the northwestern Gulf of Mexico, it has been found on *Tanacetipathes* spp. and *Phanopathes expansa* (Opresko and Cairns 1992).

The other caridean, found on *Acanthopathes thyoides*, is broken and cannot be identified definitely to species. The form of the pereopods, first antennae and rostrum are consistent with *Periclimenes antipathophilus* Spotte, Heard & Bubucis, 1994, the black coral shrimp. A photograph of our freshly collected specimen has the same color pattern as that photographed by Humann and DeLoach (2002). This species has been reported in the Bahamas, Turks and Caicos Islands, and eastern Honduras in the Caribbean on *Stichopathes gracilis* and *Stichopathes* sp. (as *Cirrhipathes* sp). It also is common at Bonaire, Netherlands Antilles (R. Heard, pers. comm.)

Also found on the antipatharians was the wing oyster *Pteria colymbus* (Röding). The wing oyster commonly occurs on gorgonians, antipatharians, or other invertebrates that project upward from the surface of reefs and is not considered to be a strict associate of antipatharians.

Multiple species of associated fauna were documented on a single black coral host. Both *Uroptychus minutus* and *Pseudopontonides principis* were found on the bottle brush shaped antipatharians *Tanacetipathes* sp. Both *Pseudopontonides principis* and *Oxynaspis gracilis* lived on sea fan antipatharian *Phanopathes expansa*.

## Discussion

Wirtz and d’Udekem-d’Acoz (2001) collected seven species of decapods from antipatharians and gorgonians at the Cape Verde Islands, none of them the same species as found in the Gulf of Mexico. These collections were made by SCUBA diving at depths of 15-30 m, considerably shallow in comparison to our collections. Of the seven species, three were found only on antipatharians, and one shrimp, *Periclimenes wirtzi* d’Udekem d’Acoz might be an obligate associate of antipatharians. Previous reports of *Periclimenes antipathophilus* indicate that it, too, is only found on antipatharians. The authors reported that one shrimp, *Pseudocoutierea wirtzi* d’Udekem d’Acoz lived in groups of ”many individuals” on gorgonians, but only solitary shrimp of this species were found on antipatharians. Because our specimens were collected by ROV, we cannot be sure if *Pseudopontonides principis* occurred in “groups” but one sample contained four individuals. Two or three individuals of *Pseudopontonides principis*, sometimes of different color patterns, have been photographed on antipatharians in the Caribbean (L. Wilk, pers.comm.). The cirripede occurred singly or in clusters of as many as 15 individuals.

To collect and observe the minute associated crustaceans of colonial cnidarians, one cannot use trawls or dredges. The smaller specimens slip through the mesh or are smashed and unrecognizable. Although the Phantom S2 is equipped with both video and still cameras, it cannot approach a black coral closely enough to see the tiny associated crustaceans. Additionally, the inability to document these interactions in situ makes defining the nature of the relationships between the associated fauna and the host difficult to determine. Although the presence of associated fauna has been documented to alter corallum or spinal morphology of the black coral host (Molodtsova and Budaeva 2007), the host fauna in this study appeared healthy and unmodified, with the exception of black coral overgrowth of *Oxynaspis gracilis*.

The majority of antipatharians in the northern Gulf of Mexico live in mesophotic or deep-sea environments, beyond the depth range of conventional SCUBA diving. Data from previous collections of antipatharians suggest that they inhabit many banks in the northern Gulf of Mexico. With proper collecting technique, it is likely that investigators will find additional specimens of the associated crustaceans and perhaps additional species.
